# Supplementary material for: Association between oral microbiome and seven types of cancers in East Asian population: a two-sample Mendelian randomization analysis
Source: Front Mol Biosci. 2023 Nov 21;10:1327893. doi: 10.3389/fmolb.2023.1327893 (PMC10702768; doi:10.3389/fmolb.2023.1327893)
Supplement: Supplementary file 1 [file DataSheet1.ZIP › Supplementary Materials/MR plots for tongue/tongue═╝/Ovarian cancer/pheno.2262_to_ovarian cancer_forest.pdf]

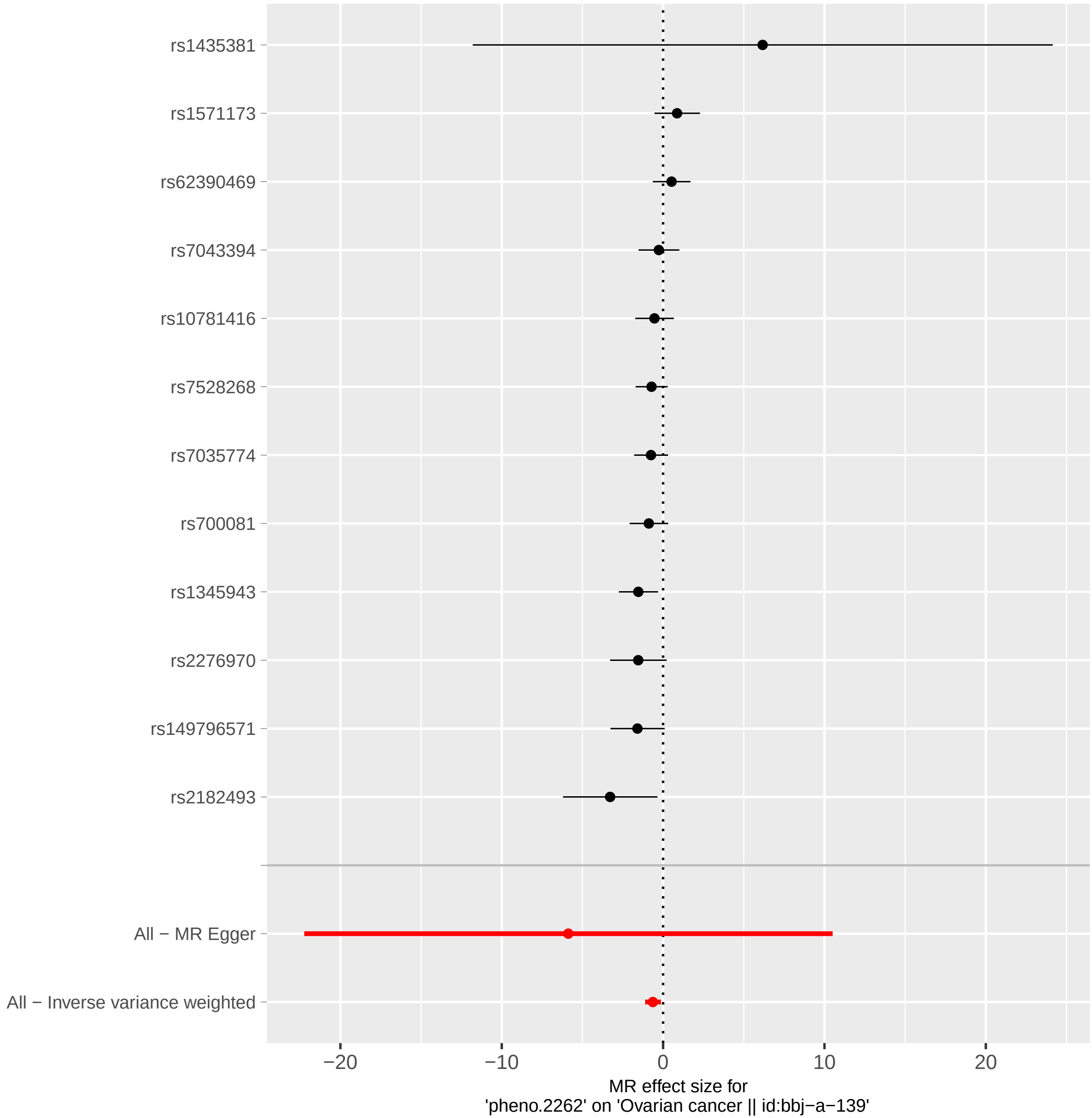

rs1435381
rs1571173
rs62390469
rs7043394
rs10781416
rs7528268
rs7035774
rs700081
rs1345943
rs2276970
rs149796571
rs2182493
All − MR Egger
All − Inverse variance weighted
−20
−10
0
10
20
MR effect size for
'pheno.2262' on 'Ovarian cancer || id:bbj−a−139'
